# A new pathological classification of intrahepatic cholangiocarcinoma according to protein expression of SSTR2 and Bcl2

**DOI:** 10.1186/s12957-021-02216-3

**Published:** 2021-05-07

**Authors:** Shoko Yamashita, Yuji Morine, Satoru Imura, Tetsuya Ikemoto, Yu Saito, Chie Takasu, Shinichiro Yamada, Kazunori Tokuda, Shohei Okikawa, Katsuki Miyazaki, Takeshi Oya, Koichi Tsuneyama, Mitsuo Shimada

**Affiliations:** 1 Department of Surgery, Tokushima University, Tokushima, 770-8503 Japan; 2 Department of Pathology and Laboratory Medicine, Tokushima University, Tokushima, 770-8503 Japan; 3 Department of Molecular Pathology, Tokushima University, Tokushima, 770-8503 Japan

**Keywords:** Bcl2, Clinicopathological characteristics, Immunohistochemistry, Prognosis, SSTR2

## Abstract

**Background:**

No universal classification method for intrahepatic cholangiocarcinoma (IHCC) has been reported based on the embryological origin of biliary epithelial cells. The aim of this study was to classify IHCC according to protein expression levels of somatostatin receptor 2 (SSTR2) and b-cell leukemia/lymphoma 2 (Bcl2) and to elucidate the clinicopathological features of each group.

**Methods:**

Fifty-two IHCC patients who underwent hepatic resection were enrolled in this study. Protein expression levels of SSTR2 and Bcl2 were examined using immunohistochemistry. Clinicopathological factors were compared between the three groups and prognostic factors were investigated.

**Results:**

The patients were divided into three groups: SSTR2 positive and Bcl2 negative (p-Group H, *n* = 21), SSTR2 negative and Bcl2 positive (p-Group P, *n* = 14), and the indeterminate group (p-Group U, *n* = 17) for cases where SSTR2 and Bcl2 were both positive or both negative. All p-Group P cases displayed curability A or B. The 5-year survival rates of p-Group H and U patients were worse than those in p-Group P. p-Group H had higher T-factor, clinical stage, and incidence of periductal infiltration than p-Group P.

**Conclusions:**

This method could be used to classify IHCC into peripheral and perihilar type by embryological expression patterns of SSTR2 and Bcl2.

## Background

Intrahepatic cholangiocarcinoma (IHCC) is a primary adenocarcinoma of the liver that arises from the intrahepatic bile ducts. It is the second most common primary hepatic tumor, after hepatocellular carcinoma (HCC), and comprises about 5–15% of total primary hepatic malignancies [1, 2]. Compared with those suffering from other malignancies, IHCC patients have an extremely poor prognosis, even if they have had curative resections performed [1, 2].

IHCC can arise from any portion of the intrahepatic biliary tree, and is classified as either perihilar or peripheral IHCC depending on where the tumor emerges [1, 2]. Tumor type was often determined based on gross appearance [3, 4]. Although most IHCC tumors are morphologically classified as adenocarcinomas, their molecular and biological features are heterogeneous. In general, the prognosis of patients with perihilar IHCC appears to be worse than those with peripheral IHCC [3, 4]. This heterogeneity may have resulted from the embryological origin of IHCC [1, 2, 5]. However, it is difficult to determine where the IHCC emerged from based only on gross findings and analysis of hematoxylin and eosin (H&E) stained histological tumor sections [6].

Previous studies have identified epidemiological and clinicopathological differences between perihilar and peripheral IHCC tumors [3–5, 7–9]. Patients who develop peripheral IHCC often suffer from chronic liver diseases such as viral hepatitis or alcoholic cirrhosis. Perihilar IHCC, however, sometimes arises in individuals with hepatolithiasis and chronic inflammation of bile ducts, including primary sclerosing cholangitis and pancreaticobiliary maljunction. Lymph node metastasis, intrahepatic metastasis, and perineural invasion occur more often in perihilar IHCC patients [3, 9]. Pathologically, higher expression levels of KRAS, S100P, and p53 are recognized in perihilar IHCC [4, 9]. In contrast, higher expression levels of isocitrate dehydrogenase 1/2 (IDH1/2) and NRAS have been observed in peripheral IHCC [4]. Further clarification of IHCC heterogeneity may provide critical information for the development of novel treatment methods. However, despite these studies, there are no universally accepted criteria for classification of an IHCC tumor as perihilar or peripheral.

Several investigators have reported that certain proteins are differentially expressed between normal small and large bile ducts in mice, rats, and humans [10–19]. γ-glutamyl transpeptidase (γ-GTP) [14], alkaline phosphatase (ALP) [14], Leucine amino peptidase (LAP) [14], Cytochrome P4502E1 [13, 15–17], secretin receptor [10, 11], Cl-/HCO3-exchanger [11, 18], and Somatostatin receptor 2 (SSTR2) [10] are all expressed in the large bile ducts but not in small ones. According to the marker of small bile duct, neural cell adhesion molecules (NCAM) and epithelial cell adhesion molecule (EpCAM) are well-known marker of cholangiolocarcinoma [2]. Although B cell leukemia/lymphoma 2 (Bcl2) is also reported to express only in bile ductule, the Bcl2 expression pattern in IHCC is still little known [12, 19]. SSTR2 belongs to the G-protein coupled receptor family and is expressed in tissues such as the cerebrum, kidney, jejunum, colon, and liver, but is most highly expressed in the pancreas [10]. Somatostatin binding to SSTR2 leads to regulation of proliferation and hormone secretion in various types of cells. Bcl2 is a protein that regulates cell death [12, 19]. Based on these findings, we hypothesized that these pathological differences between small and large normal bile ducts remain largely unchanged in malignant tissues.

In this study, we aimed to differentiate perihilar (large bile duct) and peripheral (small bile duct) carcinogenesis of IHCC through expression levels of two proteins, SSTR2 and Bcl2. We classified IHCC cases based on these criteria and then investigated the differences in clinicopathological characteristics, including prognosis, between the two groups.

## Methods

### Patient selection

Patients included in this study included 52 with IHCC, and 37 with extrahepatic cholangiocarcinoma (EHCC) as positive control was examined as control of large bile duct cancer, all of whom had undergone surgical resection at the Tokushima University Hospital between 1994 and 2017. All patients had surgical specimens available for immunohistochemistry and survived the surgery without any complications leading to mortality, such as postoperative liver failure. The IHCC patients included 34 men and 18 women ranging in age from 43 to 84 years old, with a mean age of 70.5 years. Following the Classification of Primary Liver Cancer by the Liver Cancer Study Group of Japan, T-factor was determined by number of tumors (one or more), size (no more than 2 cm in diameter), and vascular infiltration (present or absent). Tumor stage was determined by T-, N-, and M-factors. Curability of each patient was defined as A, B, or C, as follows: A: no residual tumor in stage I or II IHCC; B: no residual tumor in stage III or IV IHCC; C: residual tumor in any stage IHCC. Based on these classifications, 43 IHCC patients (82.7%) underwent resections with curability A or B. No patient received chemotherapy or irradiation before or after surgical resection. The 3- and 5-year survival rates of the IHCC patients were 40.9% and 26.8%, respectively, and the mean follow-up period was 24.7 months (range 4.4–143.8 months).

### Definition of peripheral IHCC, perihilar IHCC, and EHCC according to gross appearance

The cholangiocarcinomas were classified on the basis of finding of computed tomography (CT) into three groups: EHCC, perihilar, and peripheral IHCC. In the present study, EHCC was defined as the periductal infiltrating (PI) type of cholangiocarcinoma involving the left, right hepatic duct, and those confluences [20]. Meanwhile, mass forming (MF) or MF plus PI type of cholangiocarcinoma, located in between the right side of the umbilical portion of the left portal vein and the left side of the origin of the right posterior portal vein for tumor area, was defined as perihilar IHCC [21]. And the other MF type cholangiocarcinoma was defined as peripheral IHCC.

### Surgical procedure

All patients underwent hepatic resection. Segmentectomy involving 3 segments was performed for 6 patients, segmentectomy involving 2 segments was performed for 33 patients, and segmentectomy involving a single segment or less was performed for 13 patients. Fifteen patients underwent extrahepatic bile duct resection with hepatic resection. Lymphadectomy was performed in 29 patients. Nine patients were not performed curative resection because of the positive surgical margin or para-aortic lymph nodes metastasis.

### Immunohistochemical staining and assessment

Methods for immunohistochemical staining have been described previously [22]. Briefly, 4-μm-thick tissue sections from each sample were deparaffinized and dehydrated. Next, 0.3% hydrogen peroxide and methanol were administered to the sections for 20 min to halt peroxidase activity, followed by heat treatment. The sections were incubated with a primary rabbit polyclonal antibody to SSTR2 (NB300-157, diluted 1:200 in PBS; NOVUS Biologicals LLC, Centennial, CO, USA) and a primary mouse monoclonal antibody to Bcl2 (M088701, diluted 1:40 in PBS; Dako, Santa Clara, CA, USA) overnight at 4 °C, respectively. Sections were then treated with the secondary antibody (the EnVision^TM^+ Dual Link System-HRP, Dako) for 1 h at room temperature. SSTR2 and Bcl2 expression was evaluated by scoring the staining intensity (0, negative; 1, low; 2, medium; 3, high). Score 0 were considered as negative staining and more than 1 as positive staining. All sections of the immunostaining were evaluated by a pathologist who had no patients’ information.

### Clinicopathological analysis

The IHCC patients were divided into three groups: pathological perihilar which meant large bile duct carcinogenesis (p-Group H), SSTR2 positive, and Bcl2 negative; pathological peripheral which meant small bile duct carcinogenesis (p-Group P), SSTR2 negative, and Bcl2 positive; and unclassified (p-Group U), SSTR2 positive, and Bcl2 positive or SSTR2 negative and Bcl2 negative. Clinicopathological factors were compared between the three groups. Furthermore, prognostic factors were identified by univariate and multivariate analysis.

### Statistical analysis

All statistical analysis was performed using statistical software (JMP 13, Cary, NC, USA). Relationships between SSTR2 and Bcl2 expression and the clinicopathological variables were analyzed with one-way ANOVA analysis followed by Tukey’s test. Survival curves were generated using the Kaplan–Meier method and compared using the log-rank test. All factors found to be significant by univariate analysis were included in the Cox’s proportional hazards model of multivariate analysis to identify independent factors influencing patient survival. Statistical significance was defined as *p* < 0.05.

## Results

### Correlation between SSTR2 and Bcl2 expression and clinicopathological variables

**Fig. 1:**
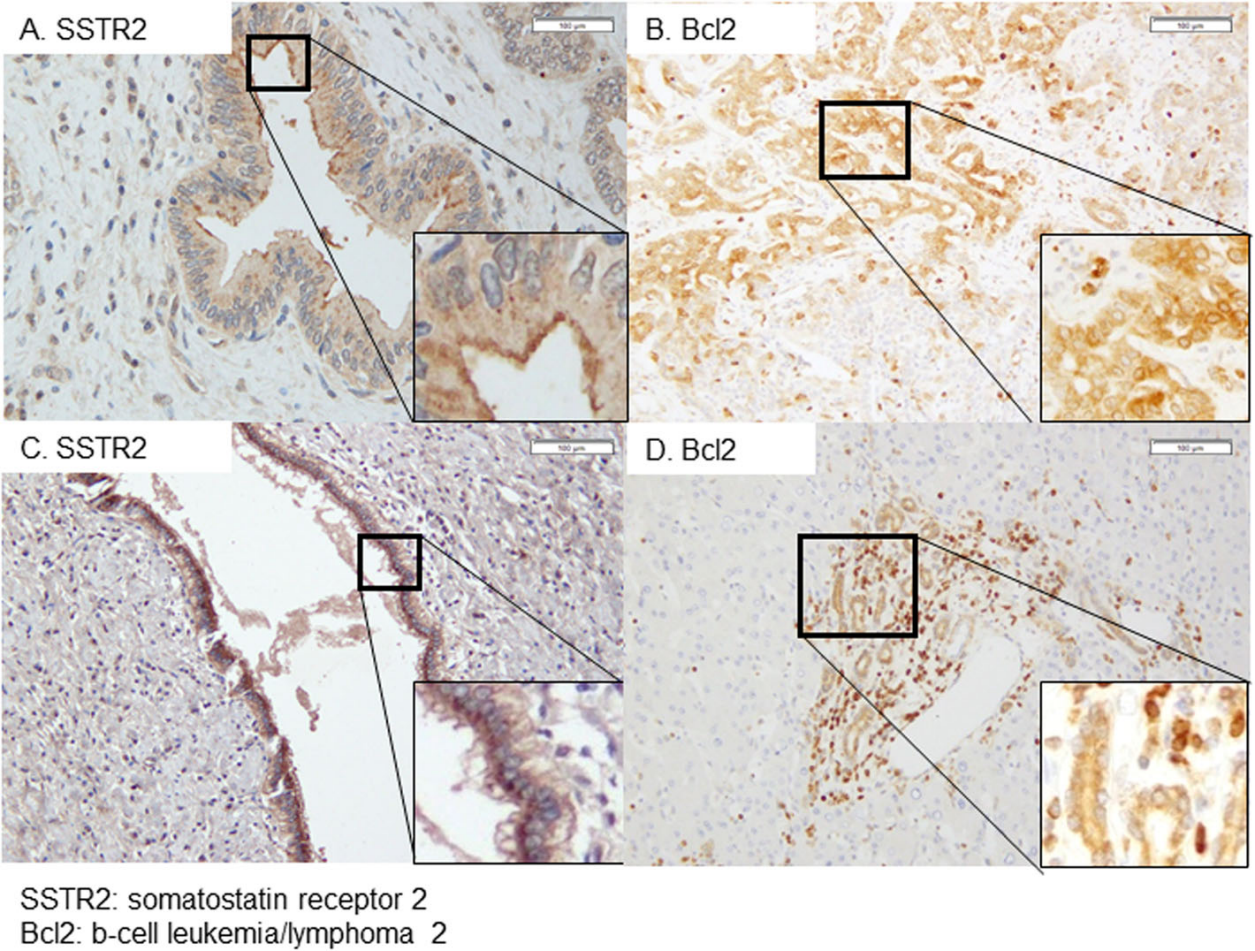
Immunostaining of IHCC tumor tissue for SSTR2 and Bcl2. **a** Expression of SSTR2 was only observed in the cell membrane of the cancer cells (× 200 magnification). **b** Expression of Bcl2 was observed in the cytoplasm of the cancer cells (× 200 magnification). **c** Expression of SSTR2 was observed in the normal large bile duct (× 200 magnification). **d** Expression of Bcl2 was observed in the normal small bile duct including bile ductule (× 200 magnification). IHCC: intrahepatic cholangiocarcinoma, SSTR2: somatostatin receptor 2, Bcl2: b cell leukemia/lymphoma 2

Tumor tissue was defined by cell staining for SSTR2 in the cell membrane (Fig. 1a) and Bcl2 in the cytoplasm (Fig. 1b). In normal part of IHCC patient’s liver, SSTR2 expression was detected in the large bile duct, while no staining in small bile duct (Fig. 1c). Bcl2 expression was detected only in small bile duct including bile ductule and interlobular bile duct (Fig. 1d). In EHCC tumors, 32 were positive for SSTR2 but negative for Bcl2, while 5 were positive for both molecules. In these five EHCC, Bcl2 was expressed, but SSTR2 was not. Only one case stained negative for both molecules. Positive SSTR2 expression in cancer cells was present in 26 out of 52 IHCC cases (50.0%) and positive Bcl2 expression in cancer cells was present in 19 (36.5%). Of 52 total IHCC patients, 21 were categorized as p-Group H (40.4%) and 14 as p-Group P (26.9%). For the remaining 17 patients in the unclassified group (p-Group U), 5 were positive for both SSTR2 and Bcl2 (9.6% of all patients), and 12 were negative for both SSTR2 and Bcl2 (23.1% of all patients).

**Fig. 2:**
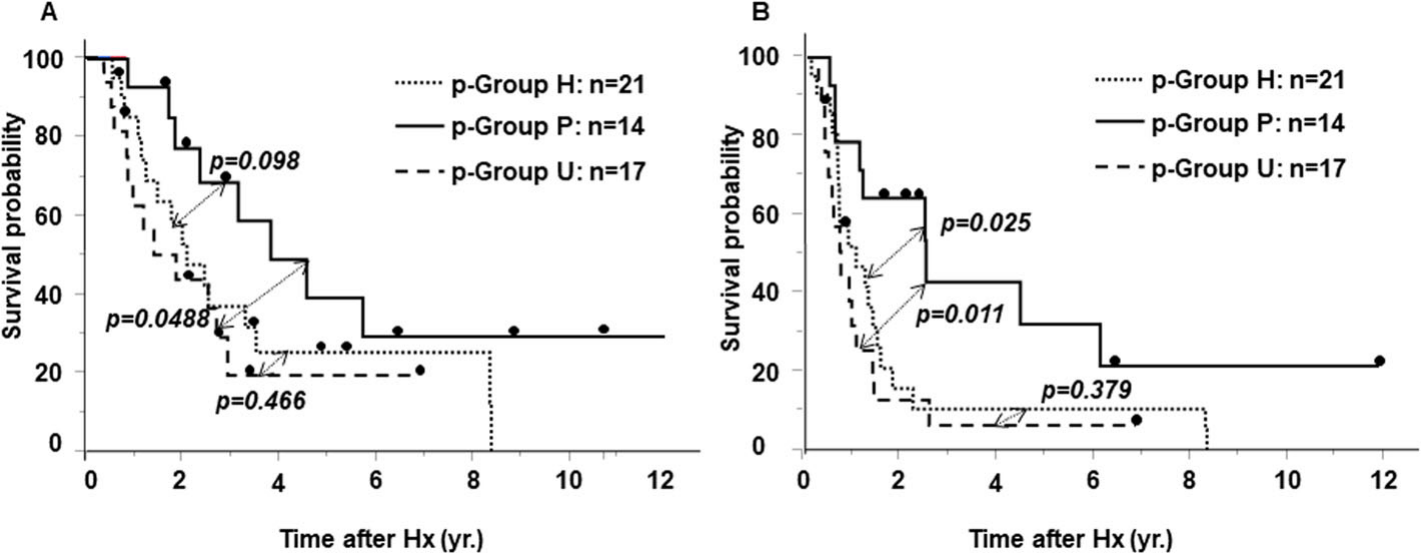
Overall survival and disease-free survival of IHCC patients classified by histological type. **a** Overall survival curves of IHCC patients. The survival of patients in the p-Group P was better than that of those in both the p-Group H and the p-Group U (*p* = 0.098, *p* < 0.05, respectively). **b** Disease-free survival curves of IHCC patients. The survival of patients in the p-Group P was significantly better than that of those in both the p-Group H and the p-Group U (*p* < 0.05). IHCC: intrahepatic cholangiocarcinoma, p-Group P: SSTR2 negative and Bcl2 positive, p-Group H: SSTR2 positive and Bcl2 negative, p-Group U: SSTR2 positive and Bcl2 positive or SSTR2 negative and Bcl2 negative

Table 1 presents a comparison of clinicopathological characteristics of IHCC patients categorized by SSTR2 and Bcl2 expression. P-Group P had a significantly better prognosis according to T classification. P-Group H and p-Group U patients had tumor infiltration into bile ducts significantly more frequently than those in p-Group P. Figure 2a shows that the overall survival of patients in p-Group P was better than that of those in p-Group H and p-Group U (*p* = 0.098, < 0.05, respectively). Similarly, Fig. 2b shows that disease-free survival of patients in p-Group P was significantly better than that of those in p-Group H and p-Group U (*p* < 0.05).

**Table 1:** Comparison of various factors among three groups of IHCC patients classified by SSTR2 and Bcl2 expression

Factors	p-Group P (*n* = 14)	p-Group H (*n* = 21)	p-Group U (*n* = 17)
Gender (M/F)	9/5	13/8	5/12
Age (years old)	68.7 ± 2.3	71.0 ± 1.9	69.6 ± 2.1
Hepatic virus infection (−/+)	9/5	15/6	11/6
Curability (A, B/C)	14/0	15/6	14/3
T (1, 2/3, 4)	9/5a	3/18a	6/11
N (0/1–3)	13/1	13/8	10/7
cStage (I, II/III, IV)	9/5a,b	2/19a	4/13b
Tumor type (MF/MF + PI)	11/3a,b	4/17a	5/12b
Tumor location (grossly peripheral/perihilar)	14/0a	16/5a,c	17/0c
Differentiation (Cholangiolo/tub1/others)	5/3/6	0/9/12	1/2/14
Tumor thrombus in the portal vein (−/+)	11/3	15/6	7/10
Tumor thrombus in the hepatic vein (−/+)	12/2	18/3	15/2
Intrahepatic metastasis (−/+)	12/2	17/4	12/5
Tumor thrombus in the bile duct (−/+)	10/4	7/14	7/10
CEA^**†**^ (ng/dL) (< 5/> 5)	11/3	13/8	11/6
CA19-9^**‡**^ (U/mL) (< 100/> 100)	10/4	7/14	7/9

^*a*^ significantly different between the p-Group P and the p-Group H

^*b*^ significantly different between the p-Group P and the p-Group U

^*c*^ significantly different between the p-Group H and the p-Group U

^**†**^ *CEA* carcinoembryonic antigen

^**‡**^ *CA19-9* carbohydrate antigen 19-9

**Fig. 3:**
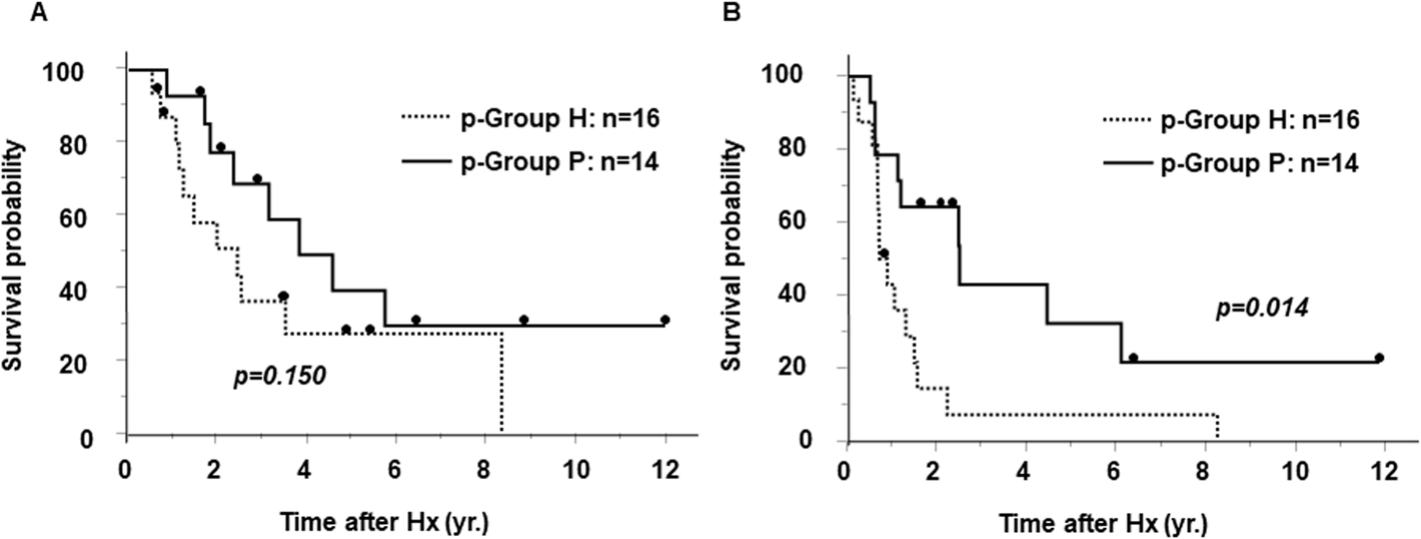
Overall survival and disease-free survival of grossly peripheral IHCC patients classified by histological type. **a** Overall survival curves of grossly peripheral IHCC patients. The survival of patients in the p-Group P was better than that of those in the p-Group H (*p* = 0.150). **b** Disease-free survival curves of grossly peripheral IHCC patients. The survival of patients in the p-Group P was significantly better than that of those in the p-Group H (*p* < 0.05). IHCC: intrahepatic cholangiocarcinoma, p-Group P: SSTR2 negative and Bcl2 positive, p-Group H; SSTR2 positive and Bcl2 negative

All five cases of perihilar IHCC based on gross classification were re-classified to p-Group H using SSTR2 and Bcl2 expressions. In addition, even in 47 cases classified as peripheral IHCC based on gross classification, we could re-classify using SSTR2 and Bcl2 expressions as follows: 14 cases were included in p-Group P with SSTR2 negative and Bcl2 positive, 16 cases in p-Group H with SSTR2 positive and Bcl2 negative, and 17 cases in p-Group U. Comparing these 14 individuals in p-Group P with the 16 in p-Group H, those in p-Group H had lower curability (*p* = 0.031), a higher T-factor (*p* = 0.005), higher clinical stage (*p* = 0.001) (Table 2), higher incidence of periductal infiltration (*p* = 0.005), and worse prognosis according to disease-free survival (*p* = 0.014) (Fig. 3). Grossly classified peripheral IHCC included more p-Group H than p-Group P, and the p-Group H patients did significantly worse.

**Table 2:** Comparison of various factors among two groups of grossly peripheral IHCC patients classified by SSTR2 and Bcl2 expression

Factors	p-Group P (*n* = 14)	p-Group H (*n* = 16)	*p* value
Gender (M/F)	9/5	12/4	0.405
Age (years old)	68.7 ± 2.4	71.3 ± 2.2	0.221
Hepatic virus infection (−/+)	9/5	12/4	0.405
Curability (A, B/C)	14/0	10/6	0.031
T (1, 2/3, 4)	9/5	2/14	0.005
N (0/1–3)	13/1	10/6	0.061
cStage (I, II/III, IV)	9/5	1/15	0.001
Tumor type (MF/MF + PI)	11/3	4/12	0.005
Differentiation (Cholangiolo/tub1/others)	5/3/6	0/8/8	0.009
Tumor thrombus in the portal vain (−/+)	11/3	14/2	0.426
Tumor thrombus in the hepatic vain (−/+)	12/2	14/2	0.482
Intrahepatic metastasis (−/+)	12/2	14/2	0.482
Tumor thrombus in the bile duct (−/+)	10/4	7/9	0.391
CEA† (ng/dL) (< 5/> 5)	11/3	11/5	0.426
CA19-9‡ (U/mL) (< 100/> 100)	10/4	7/9	0.972

† *CEA* carcinoembryonic antigen

‡ *CA19-9* carbohydrate antigen 19-9

### Representative cases

**Fig. 4:**
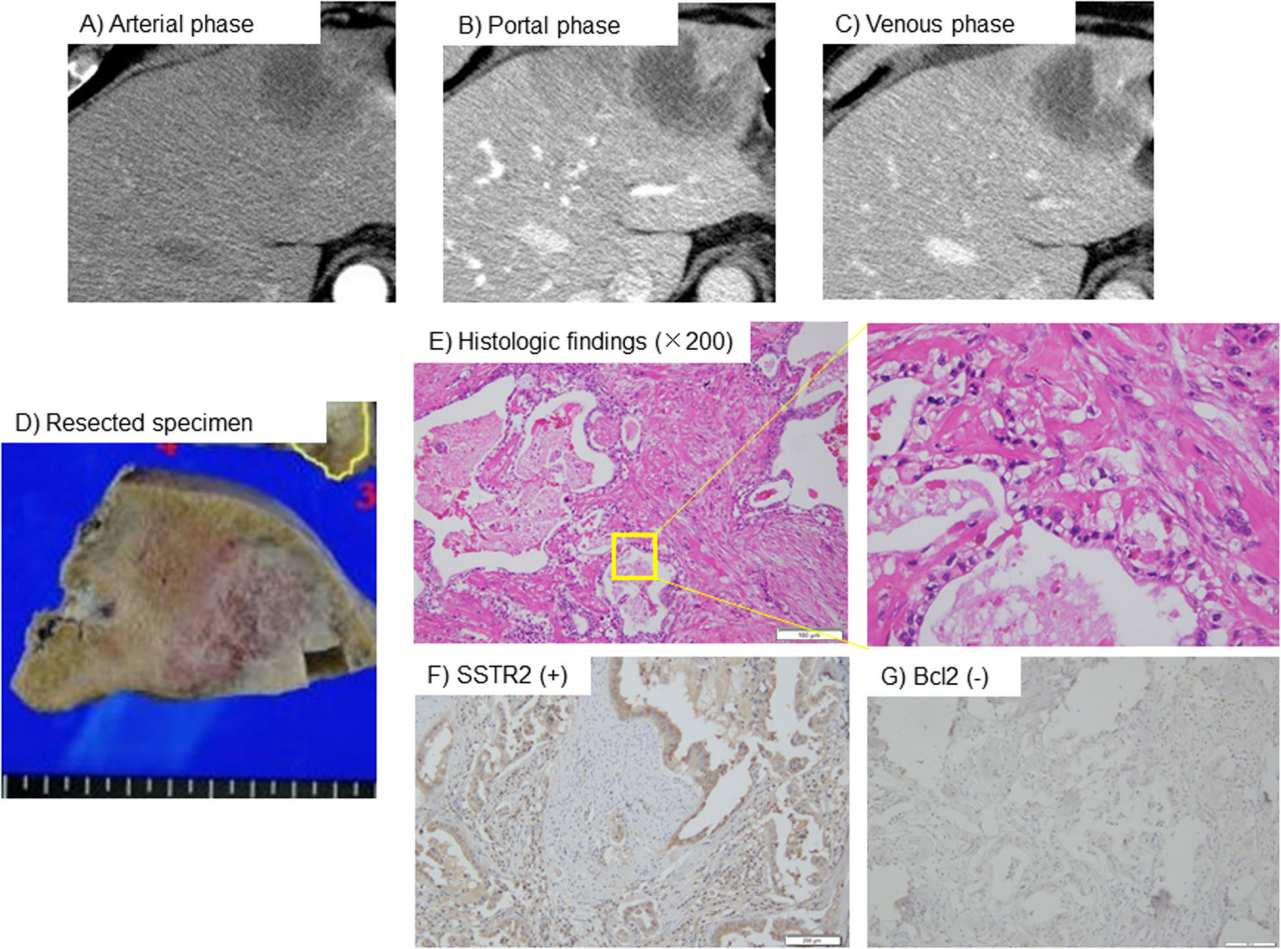
Representative images of gross and histological findings in IHCC tumors. **a**–**c** CT images showed the presence of a 5.5 cm, low density mass in the S3 segment of the liver in all phases. **d** The resected specimen showed the presence of a nodular tumor in the S3 segment of the liver. This tumor was consistent with the mass-forming type. **e** Histologic findings (× 100 magnification) revealed that the tumor consisted of duct-forming, abundant fibrotic stroma. The cancer cells had a clear cytoplasm. **f** SSTR2 expression can be seen in the cell membrane of IHCC tumor cells (× 100 magnification). **g** IHCC cancer cells were negative for Bcl2 (× 100 magnification). IHCC: intrahepatic cholangiocarcinoma, CT: computed tomography. SSTR2: somatostatin receptor 2, Bcl2: b-cell leukemia/lymphoma 2

According to typical histological findings described by Akita and Liau [6, 9], perihilar IHCC consists of duct-forming and tall columnar tumor cells, as well as an abundant fibrotic stroma [6, 9]. This type of tumor has a clear cytoplasm and tubular components similar to reactive bile ductules at the tumor-liver interface of the invasive front. In contrast, peripheral IHCC consists of cuboidal to low columnar cells, which form irregularly anastomosing tubular architecture with scant mucin. This type of IHCC typically has a hepatoid appearance. Figure 4 shows a representative case. According to the CT images and gross appearance, the tumor appeared to be located in the periphery of the liver (Fig. 4a–d). However, morphologically, the tumor consisted of duct-forming, abundant fibrotic stroma and little tubular architecture and cancer cells had a clear cytoplasm, which suggested it to be the perihilar type (Fig. 4e). Additionally, immunohistochemical analysis indicated that SSTR2 was expressed in the tumor, but Bcl2 was not (p-Group H) (Fig. 4f, g). Considering these observations, this tumor was classified as embryological perihilar immunohistologically, but as peripheral based on its gross appearance.

### Relationship between the bile duct and IHCC tumor according to 3D imaging

**Fig. 5:**
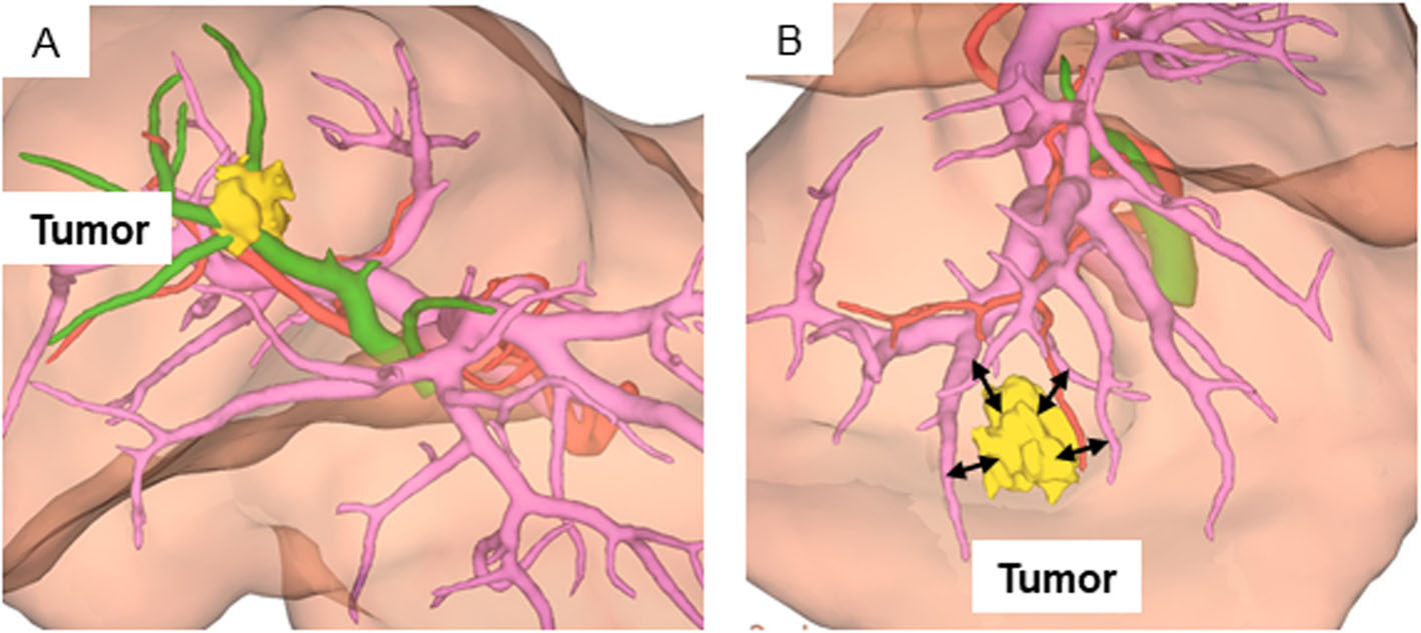
3D image of the IHCC tumor. **a** 3D image showing a case with the IHCC tumor connected to the vessels. **b** 3D image showing a case with the IHCC tumor not connected to the vessels. IHCC: intrahepatic cholangiocarcinoma

Figure 5 shows the relationship between the bile duct and IHCC tumor according to 3D imaging. The CT image was reconstructed using SYNAPSE VINCENT® (Fujifilm, Japan), and we can identify larger vein and artery than the subsegmental branch. The distance between tumor and vessels was evaluated using 3D imaging for 17 cases of IHCC (p-Group H: 10 cases, p-Group P: 7 cases). The tumor contacted the bile duct in all cases in p-Group H (Fig. 5a). However, in p-Group P, the tumor contacted the bile duct in only four of the seven cases (Fig. 5b).

### Analysis of prognostic factors

Table 3 shows the results of the univariate analysis of overall survival. The stage, curability, T-factor, lymph node metastasis, tumor thrombus in the portal vein, intrahepatic metastasis, and carbohydrate antigen 19-9 (CA19-9) were found to be significant prognostic factors for overall survival, as well as the SSTR2 and Bcl2 expression pattern (p-Group H and U).

**Table 3:** Analysis of overall survival in IHCC patients

Univariate analysis				Multivariate analysis			
Factors	*n*/*n*	5-year survival (%)	*p* value	Factors	Hazard ratio	95% C.I.	*p* value
Curability (A, B/C)	43/9	32.3/0.00	0.003	Curability (A, B/C)	1.634	0.566–4.306	0.348
T (1, 2/3, 4)	18/34	57.0/10.4	< 0.001	T (1, 2/3, 4)	2.847	0.944–9.313	0.064
N (0/1–3)	36/16	36.5/6.73	0.001	N (0/1–3)	3.091	1.348–7.002	0.008
cStage (I, II/III, IV)	15/37	61.9/13.0	< 0.001				
Tumor type (MF/MF + PI)	20/32	38.6/19.2	0.163				
Tumor location (grossly peripheral/perihilar)	47/5	27.7/20.0	0.667				
Differentiation (Cholangiolo/tub1/others)	6/14/32	40.0/17.9/30.8	0.235				
SSTR2^**†**^, Bcl2^**‡**^ expression (p-Group H + U/p-Group P)	38/14	22.6/39.3	0.046	SSTR2, Bcl2 (p-Group H + U/p-Group P)	1.055	0.370–2.880	0.917
Tumor thrombus in the portal vein (−/+)	33/19	35.7/9.40	0.001	Tumor thrombus in the portal vein (−/+)	2.237	0.963–5.383	0.061
Tumor thrombus in the hepatic vein (−/+)	45/7	25.9/28.6	0.660	Intrahepatic metastasis (−/+)	2.049	0.712–5.474	0.176
Intrahepatic metastasis (−/+)	44/8	30.3/12.5	0.030				
CA19-9^**§**^ (< 100/> 100)	24/27	48.3/6.00	0.008	CA19-9 (< 100/> 100)	1.485	0.591–3.793	0.400
CEA^||^ (< 5/> 5)	37/15	27.4/26.6	0.810				
Surgical procedure (Partial resection/segmentectomy / lobectomy)	5/8/39	30.0/38.1/25.5	0.627				
Lymphadenectomy (+/−)	29/23	17.1/40.4	0.1027				

^**†**^ *SSTR2* somatostatin receptor 2

^**‡**^ *Bcl2* bcell leukemia/lymphoma 2

^**§**^ *CA19-9* carbohydrate antigen 19-9

^||^ *CEA* carcinoembryonic antigen

Table 3 also shows the results of the multivariate analysis of overall survival. Lymph node metastasis (hazard ratio = 3.091) was identified as independent prognostic factors. However, the SSTR2 and Bcl2 expression pattern was not found to be an independent prognostic factor.

## Discussion

In this study, we examined the expression of SSTR2 and Bcl2 in IHCC tumors. The data suggested that the expression pattern of these molecules may correlate with the location of the tumors. Our embryological classification approach may be considered reasonable clinicopathologically and useful for the classification of cases that are difficult to identify histologically.

Our study is based on previous investigations that have described the heterogeneity of normal bile ducts according to their location [10–19]. Currently, there are no criteria for classification of these IHCC tumors based on any molecules that potentially display differential expression when compared with normal bile ducts. As mentioned above, we believe that changes to pathological characteristics will inevitably occur when a bile duct develops into a tumor, which potentially threaten diagnostic precision. For example, S100 protein (S100) are expressed both in tumor tissue and in normal bile ducts [8]. However, there was a report that higher expression levels of S100P were detected in cholangiocarcinoma compared with the benign biliary strictures [23]. Additionally, mucin production also varies during each stage of carcinogenesis [8, 24]. EpCAM and NCAM are well known marker of cholangiolocellular carcinoma. However, EpCAM is broadly expressed in both large and small bile ducts [25]. NCAM was reported to be expressed in the reactive proliferating bile ductules of the diseased livers. Therefore, EpCAM and NCAM could not be the specific marker of small bile duct including septal bile duct, interlobular bile duct and bile ductule [26]. To address this effect, we selected SSTR2 and Bcl2. To best of our knowledge, there is no report suggesting that the expression of SSTR2 or Bcl2 relates to tumor malignancy, and we believe that this new method may be useful for IHCC classification. According to numerous studies, the prognosis of perihilar IHCC patients is worse than that of peripheral IHCC patients, and there is also an increased incidence of lymph node metastasis in these individuals [3, 4, 6, 7]. These facts demonstrate why careful and accurate classification of IHCC type is necessary.

Previous studies have used various criteria to classify IHCC tumors, including gross appearance and imaging findings [3, 4], histopathologic appearance [3, 4, 6, 9], and immunophenotypes [7]. Although the gross appearance and imaging classification approach is considered to be the most practical, some tumors derived from the peripheral duct can move into the hepatic portal region, making it difficult to determine the origin of the IHCC using this method. Aishima et al. classified IHCC tumors that were smaller than 5 cm in diameter based on gross appearance, defining perihilar tumors as those involving segmental or larger bile ducts and peripheral tumors as those only affecting smaller ducts [3]. Ruzzenente et al. also classified IHCC on the basis of gross appearance, specifically by the location of the center of the tumor. However, these criteria could not specifically identify the origin of larger tumors. When this occurred, evaluation was also performed by a pathologist and radiologist [3, 4]. These studies suggest that classifying an IHCC tumor based only on gross appearance is a difficult task [4].

Some reports have described histopathological classifications of IHCC tumors [3, 4, 6, 9]. A publication by Liau et al. stated that the perihilar type is composed of tall columnar cells, while the peripheral type consists of cuboidal to low columnar cells [9]. Akita et al. reported that perihilar tumors display ductal adenocarcinoma, but perihilar ones have tubular components in the central parts of the tumor. Although the authors could classify these IHCC tumors using histopathological criteria, reproducibility is still an issue. They reported that their standard had a kappa value of less than 0.6, and diagnosing the origin of IHCC is a difficult task, even for some pathologists [6].

Immunohistochemical classification, such as our use of SSTR2 and Bcl2 expression, is a relatively simple method for both pathologists and non-pathologists alike. However, some cases still have unclear results and are categorized as indeterminate. In fact, our study had 17 indeterminate cases that were placed in the unclassified group (p-Group U)—tumors that were positive for both SSTR2 and Bcl2 or negative for both SSTR2 and Bcl2. Although characteristics of p-Group U is similar to p-Group H according to prognostic factor including T classification and the tumor infiltration into bile duct, these 17 cases were difficult to classify into p-Group P or p-Group H based on our method. Only 35 cases (67%) of IHCC could be reliably classified as perihilar or peripheral, which is the most serious limitation of our study. It is possible that certain characteristics, including expression of several molecules, are altered when normal tissue becomes malignant. In contrast, Hayashi’s criteria for classification utilize a scoring system which is based on the mucin productivity and immunophenotype of the cells (S100P, N-cadherin, and neural cell adhesion molecule (NCAM)). The authors reported that 98 of 102 cases (96%) could be classified into two types of IHCC [7]. Compared with our results, this rate is extremely high. However, scoring the cases after performing immunostaining many times is complicated.

Our results suggest that pathological classification may partially correlate with gross classification. A limitation of gross classification using 3D imaging is that large tumors often appear to be connected to the bile duct in both perihilar and peripheral IHCC. However, 3D imaging may be effective when used in combination with the pathological classification method.

## Conclusions

Our work demonstrates a novel approach to classify IHCC tumors as peripheral or perihilar based on the expression patterns of SSTR2 and Bcl2 embryologically. Yet, about 30% of cases could not be classified by this method. Further research is needed to determine whether adding more molecules to the expression analysis will improve the success of our IHCC classification technique.

## Data Availability

The datasets generated and analyzed during the current study are not publicly available to protect the individual privacy but are available from the corresponding author on reasonable request.

## Abbreviations

IHCC: Cholangiocarcinoma
SSTR2: Somatostatin receptor 2
Bcl2: B cell leukemia/lymphoma 2
HCC: Hepatocellular carcinoma
H&E: Hematoxylin and eosin
IDH 1/2: Isocitrate dehydrogenase 1/2
γ-GTP: γ-glutamyl transpeptidase
ALP: Alkaline phosphatase
LAP: Leucine amino peptidase
CT: Computed tomography
CA19-9: Carbohydrate antigen 19-9
NCAM: Neural cell adhesion molecules
EpCAM: Epithelial cell adhesion molecule
